# Biological and Pathophysiological Mechanisms of Postoperative Complications Affecting Long-Term Survival: A Narrative Review

**DOI:** 10.7759/cureus.112090

**Published:** 2026-07-05

**Authors:** Laurence Weinberg, Junyan Zhao, Luca Petterlin, Emasha Seneviratne, Rebecca Wang, Jemin Suh, Parthiv Gaykar, Tareeque Knight, Warren Lee, Dong-Kyu Lee, Nattaya Raykateeraroj

**Affiliations:** 1 Department of Anesthesia, Austin Health, Melbourne, AUS; 2 Department of Critical Care, The University of Melbourne, Melbourne, AUS; 3 Department of Anesthesiology and Pain Medicine, Dongguk University Ilsan Hospital, Goyang, KOR; 4 Department of Anesthesiology, Faculty of Medicine, Siriraj Hospital, Mahidol University, Bangkok, THA

**Keywords:** immune dysregulation, inflammation, long-term survival, neuroendocrine stress, postoperative complications

## Abstract

Postoperative complications are common after major surgery and are consistently associated with poorer long-term survival, yet the biological mechanisms underpinning this relationship remain incompletely understood. This narrative review aimed to synthesise clinical and mechanistic evidence linking postoperative complications to long-term mortality and major adverse outcomes.

We conducted a narrative review with structured search elements using Ovid MEDLINE and Embase (Excerpta Medica dataBASE) from database inception to November 30, 2025, supplemented by Google Scholar searching, citation tracking, and expert-identified references; Ovid MEDLINE was additionally updated through March 31, 2026. Eligible evidence included randomised and non-randomised trials, observational cohort studies, systematic reviews, and mechanistic studies, including translational, in vitro, and in vivo investigations, that reported either long-term outcomes after postoperative complications or biological pathways linking postoperative complications with later adverse outcomes. Data were synthesised descriptively and organised into predefined mechanistic domains; formal meta-analysis and formal risk-of-bias assessment were not undertaken because of substantial clinical and methodological heterogeneity and the mechanistic purpose of the review.

Of 1,329 database records and 163 additional records identified through supplementary methods, 84 studies met the inclusion criteria and were incorporated into the review. Across both oncological and non-oncological surgery, postoperative complications were consistently associated with impaired long-term survival. Four recurrent mechanistic domains emerged: persistent systemic inflammation, exaggerated neuroendocrine stress responses, perioperative immune dysregulation, and disruption to adjuvant treatment pathways in selected cancer populations. Collectively, the available evidence suggests that postoperative complications may act not only as markers of illness severity but also as biological amplifiers with the potential to influence long-term prognosis, although the degree of causality remains uncertain and much of the mechanistic evidence is translational or preclinical.

Postoperative complications appear to worsen long-term survival through interacting inflammatory, neuroendocrine, and immune pathways, with additional contributions from disrupted oncological treatment in some cancers. Reframing complications as biologically consequential perioperative events highlights the perioperative period as a potentially important therapeutic window and supports future biomarker-rich cohort studies and interventional trials targeting these pathways.

## Introduction and background

Postoperative complications are common after major surgery and are a major cause of morbidity, mortality, prolonged hospital stay, and healthcare cost [1-4]. An estimated 313 million operations are performed worldwide each year, and approximately one in five patients undergoing major surgery will experience a postoperative complication [1,2]. Although these events are often considered perioperative outcomes, growing evidence suggests that their effects extend far beyond the immediate postoperative period [5,6].

Postoperative complications have consistently been associated with reduced long-term survival after both oncological and non-oncological surgery [6,7]. In some cohorts, they appear to be more strongly associated with long-term mortality than preoperative risk factors or intraoperative events. Patients who develop complications such as pneumonia, deep wound infection, or pulmonary embolism have worse longer-term outcomes even after apparent recovery from the initial postoperative episode [5-7].

However, the biological basis of this association remains poorly integrated. Existing literature has largely described epidemiological associations, whereas the mechanistic pathways linking postoperative complications to later mortality have received less attention [5-7]. Emerging evidence implicates persistent systemic inflammation, exaggerated neuroendocrine stress responses, perioperative immune dysregulation, and delays to adjuvant therapy in selected cancer populations [8-29]. These observations suggest that postoperative complications may act not only as markers of severe illness but also as biological amplifiers with the potential to shape long-term cardiovascular and oncological outcomes.

This narrative review synthesises clinical, translational, and experimental evidence to examine the biological and pathophysiological mechanisms through which postoperative complications may impair long-term survival and to highlight implications for perioperative research and practice.

Methods

Aim

This study is a narrative review informed by structured database searches. The primary objective was to synthesise published evidence on the biological and pathophysiological mechanisms by which postoperative complications may be associated with reduced long-term survival. Secondary objectives were to identify implications for perioperative practice and to highlight priorities for future mechanistic and interventional research. A narrative approach was selected because the question spans heterogeneous evidence types, including observational clinical studies, translational biomarker studies, experimental models, and mechanistic oncology literature, which are not readily amenable to quantitative pooling within a single meta-analytic framework.

Search Strategy and Selection Criteria

Structured searches were undertaken in Ovid MEDLINE and Embase (Excerpta Medica dataBASE) from database inception to November 30, 2025. In addition, the Ovid MEDLINE search was updated through March 31, 2026, to identify recently published studies relevant to the mechanistic focus of the review. The search strategy combined three concept domains, namely, postoperative complications, long-term survival, and mechanistic pathways, using both controlled vocabulary terms and free-text keywords, including Medical Subject Headings (MeSH) and Emtree terms where appropriate. Complete database-specific search strategies are provided in the Appendices. Supplementary searching was undertaken using Google Scholar, backward and forward citation tracking of landmark studies, and expert-identified seed references. Eligible studies included randomised and non-randomised controlled trials, prospective and retrospective observational studies, cohort studies, systematic reviews, meta-analyses, and mechanistic studies including translational, in vitro, and in vivo investigations. Studies were included if they reported long-term survival or other major adverse long-term outcomes after postoperative complications or provided mechanistic data linking postoperative complications with later adverse outcomes. Conference abstracts without full text, case reports, duplicate publications, and non-English publications were excluded. The focused Ovid MEDLINE update did not identify any additional eligible studies beyond those already captured in the original database search.

Study Selection

Titles and abstracts were screened for relevance, followed by full-text review of potentially eligible articles. Screening and full-text eligibility assessment were undertaken by two reviewers (LW, JZ) using predefined eligibility criteria, with disagreements resolved by discussion and, where required, adjudication by a senior author (NR). Given the narrative and mechanistic purpose of the review, study selection was designed to identify clinically and biologically informative literature rather than to support pooled quantitative effect estimation alone.

Data Extraction and Synthesis

Data were extracted using a structured template that captured study design, surgical population, complication type, complication severity classification, duration of follow-up, long-term outcomes, and mechanistic findings. Where reported, complication severity was classified according to frameworks such as the Clavien-Dindo classification, Comprehensive Complication Index, infectious complication definitions, or study-specific severity criteria. Because definitions of postoperative complications varied across the literature, complications were interpreted within the context of each individual study and then grouped descriptively into clinically relevant categories for synthesis, including infectious versus non-infectious complications and minor versus major complications where possible.

Findings were synthesised descriptively and organised into four recurrent mechanistic domains identified across the literature: persistent systemic inflammation, neuroendocrine stress responses, perioperative immune dysregulation, and disruption to adjuvant treatment pathways in selected oncological settings. Within each domain, we distinguished between evidence derived from clinical cohort studies, translational biomarker studies, and preclinical experimental studies to better reflect differences in evidentiary strength. Meta-analysis was not undertaken because of substantial heterogeneity in study designs, surgical populations, definitions of postoperative complications, complication severity, mechanistic endpoints, long-term outcome measures, and follow-up duration. Formal risk-of-bias assessment and formal evidence grading were not performed because a substantial proportion of the included evidence was mechanistic or preclinical and because the aim of the review was integrative biological synthesis rather than quantitative effect estimation. These methodological choices are acknowledged as limitations and should be considered when interpreting the findings.

## Review

Results

Search Results and Study Selection

**Figure 1:**
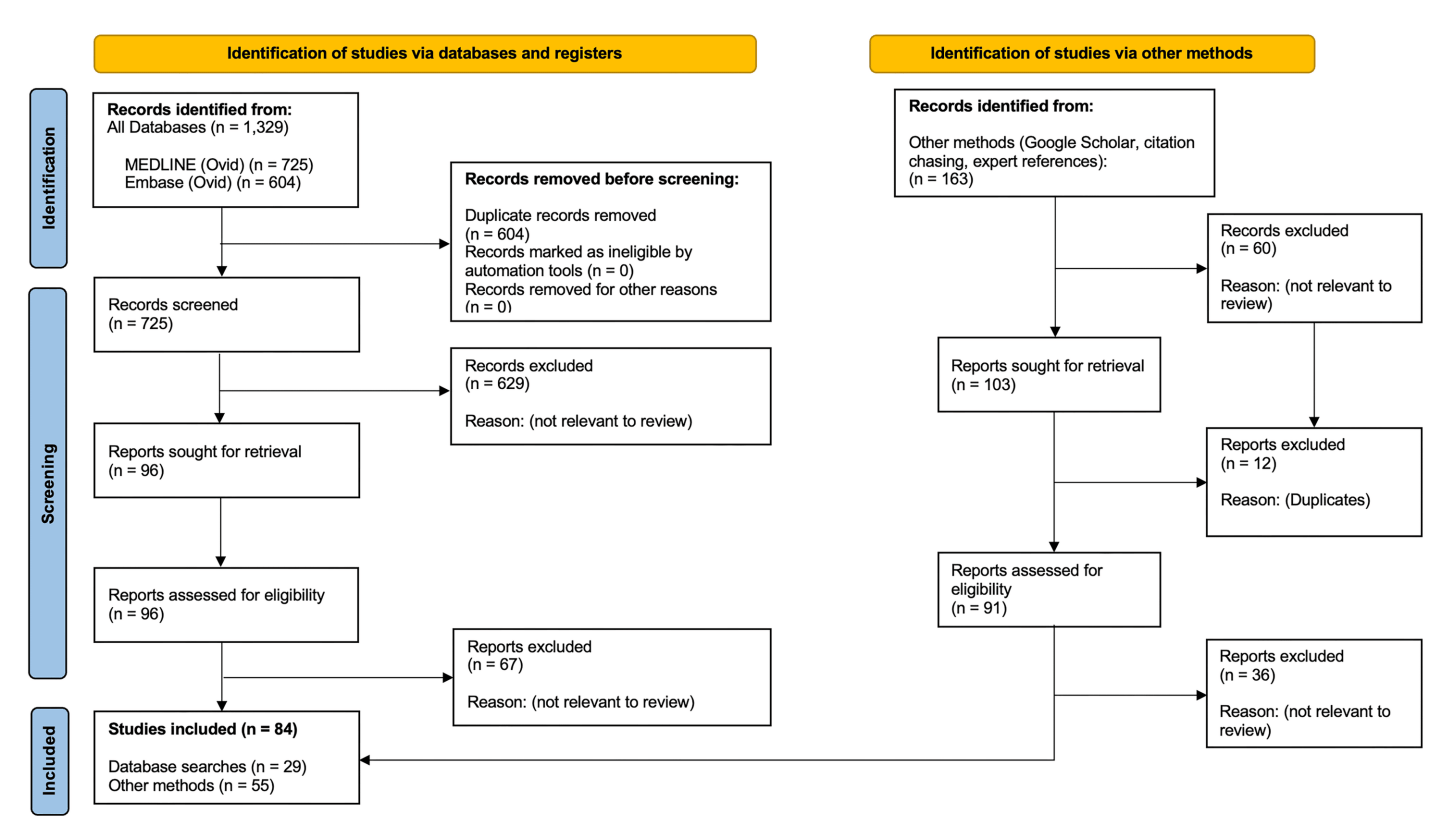
PRISMA flow diagram Embase: Excerpta Medica dataBASE; PRISMA: Preferred Reporting Items for Systematic Reviews and Meta-Analyses

Across database searches, 1,329 records were identified, comprising 725 citations from Ovid MEDLINE and 604 from Embase. After the removal of duplicates, 725 unique records underwent title and abstract screening, of which 629 were excluded. Ninety-six reports were retrieved for full-text assessment, and 67 were excluded, yielding 29 eligible studies from database searches. In parallel, 163 additional records were identified through supplementary methods, including Google Scholar, citation tracking, and expert suggestion. After screening and full-text assessment, 55 eligible studies from supplementary sources were included. In total, 84 studies met the inclusion criteria and were incorporated into the review. The study selection process is summarised in Figure 1. The focused Ovid MEDLINE update through March 31, 2026, did not yield any additional eligible studies.

Characteristics of the Included Studies

The included literature comprised a heterogeneous evidence base spanning oncological and non-oncological surgery, observational clinical cohorts, systematic reviews, translational biomarker studies, and preclinical experimental investigations. Complication definitions and severity classifications varied across studies and included infectious complications, cardiopulmonary complications, anastomotic leak, wound complications, and broader major postoperative morbidity, variably defined using the Clavien-Dindo classification, Comprehensive Complication Index, or study-specific criteria. Follow-up duration and outcome measures also varied substantially, ranging from recurrence-focused oncological outcomes to all-cause mortality and organ-specific adverse events. The evidence supporting the proposed mechanisms linking postoperative complications with long-term outcomes is summarised in Table 1.

**Table 1:** Summary of evidence supporting the proposed mechanisms linking postoperative complications with long-term outcomes The table reflects the dominant relevance of each evidence stream; individual studies may inform more than one pathway. CRP: C-reactive protein; IL: interleukin; PGE2: prostaglandin E2; COX-2: cyclooxygenase-2

Mechanistic domain	Predominant evidence base	Main settings and exposures	Long-term outcomes assessed	Interpretation and limitations
Persistent systemic inflammation	Clinical cohorts; translational biomarker studies; mechanistic reviews and experimental models	Major non-cardiac, cardiac, abdominal, and colorectal surgery; critical illness and cancer biology. Infection/sepsis, systemic inflammatory response, IL-6/CRP elevation, myocardial acidosis, COX-2/PGE2, and growth factor signalling	Long-term survival, cardiovascular events, organ injury, tumour growth, recurrence, and metastasis	Clinical associations are predominantly observational; perioperative mechanistic links are heterogeneous and are often indirect
Neuroendocrine stress responses	Mechanistic reviews; in vitro, animal, and translational studies; limited early clinical/interventional evidence	Cancer surgery and tumour models; surgical stress, catecholamine/β-adrenergic activation, sympathetic innervation, and lymphatic remodelling	Tumour cell proliferation, invasion, angiogenesis, lymphatic trafficking, and metastatic susceptibility	The evidence base is largely preclinical; the magnitude of effect in specific clinical surgical populations remains uncertain
Perioperative immune dysregulation	Translational immune profiling; mechanistic reviews; experimental intervention studies; limited clinical cohort evidence	Major surgery, trauma, and cancer surgery. Natural killer- and CD8-positive T-cell suppression, regulatory T-cell expansion, macrophage polarisation, and altered innate immune responses	Delayed recovery, impaired immune competence, metastatic susceptibility, and cancer recurrence	The biological signal is coherent, but the independent causal contribution beyond baseline disease and surgical stress is uncertain
Disruption to adjuvant treatment pathways	Systematic reviews/meta-analyses and retrospective or population-based clinical cohorts	Surgery for colorectal liver metastasis, colorectal, lung, gastric, breast, or laryngeal cancer, or sarcoma. Anastomotic leak, infection, and major postoperative complications	Delayed or omitted chemotherapy/radiotherapy, recurrence, disease-specific survival, and overall survival	This pathway is context-dependent; effects vary by tumour type, stage, treatment regimen, and complication severity

The detailed study-level evidence base of the included studies [1-84], including contextual studies of global surgery, complication classification, and outcome measurement, is provided in Table 2.

**Table 2:** Characteristics of the included studies organised by primary mechanistic relevance Studies are grouped according to the primary mechanistic pathway most directly represented by their reported findings. Categories are not mutually exclusive. "Not reported" denotes that a severity classification was not available from the citation or source material used for this narrative review. CCI: Comprehensive Complication Index; COX-2: cyclooxygenase-2; CRP: C-reactive protein; IL: interleukin; MMP: matrix metalloproteinase; PGE2: prostaglandin E2; SIRS: systemic inflammatory response syndrome; VEGF: vascular endothelial growth factor; VA/NSQIP: VA National Surgical Quality Improvement Program; TLR: Toll-like receptor; c-FLIP: cellular FLICE-like inhibitory protein; hs-CRP: high-sensitivity C-reactive protein; hs-cTnT: high-sensitivity cardiac troponin T

Study	Design and setting	Complication/exposure	Severity definition	Follow-up	Long-term outcome(s)/contribution
Contextual and classification evidence (not assigned to a mechanistic domain) (n=5)					
Weiser et al. (2015) [1]	Global modelling/ecological estimate; global surgery volume	Not applicable	Not applicable	Not applicable (cross-sectional 2012 volume estimate)	Global surgical volume; no patient survival outcome
Pearse et al. (2016) [2]	Prospective international cohort; elective inpatient non-cardiac surgery	Postoperative complications	Protocol-defined morbidity; formal severity classification not reported	7 days (in-hospital)	Early postoperative morbidity and 7-day/in-hospital mortality; no long-term survival
Dindo et al. (2004) [3]	Classification development/validation study; general surgery	Postoperative complications	Clavien-Dindo	30-day postoperative complications	Classification performance; no long-term survival
Vonlanthen et al. (2011) [4]	Retrospective cohort/cost analysis; major surgical procedures	Postoperative complications	Clavien-Dindo	Index admission	Costs and resource use; no long-term outcome
Abbott et al. (2017) [6]	National ecological study; UK hospital procedures	Not applicable	Not applicable	Annual activity data	Procedure frequency; no patient-level long-term outcome
Persistent systemic inflammation (n=34)					
Khuri et al. (2005) [5]	Prospective multicentre cohort; major surgery (VA/NSQIP)	Postoperative complications	VA/NSQIP morbidity definitions	Up to 8 years	Long-term survival
Fowler et al. (2022) [7]	Secondary analysis of pooled prospective cohorts; elective non-cardiac surgery	Postoperative complications	Study-specific morbidity definitions	1 year	All-cause mortality
Ni Choileain and Redmond (2006) [8]	Mechanistic review; surgery/trauma	Surgical stress response	Not applicable	Not applicable	No long-term clinical outcomes (mechanistic review)
Baigrie et al. (1992) [10]	Prospective clinical physiology study; major surgery	Cytokine response	Not applicable	Early postoperative period	No long-term clinical outcomes (short-term cytokine study)
Bain et al. (2023) [11]	Prospective cohort; major abdominal surgery	Postoperative systemic inflammation	Not reported	Early postoperative period	Patient-centred recovery outcomes
Bain et al. (2023) [12]	Narrative review; major surgery	Inflammatory dysregulation/corticosteroids	Not applicable	Not applicable	No long-term clinical outcomes (narrative review)
Lahiri et al. (2016) [13]	Prospective translational cohort; major abdominal surgery	SIRS; TLR4/TLR5 upregulation	SIRS criteria	Early postoperative period	SIRS/clinical recovery
Held et al. (2017) [15]	Secondary analysis of randomised controlled trial cohort; stable coronary heart disease	IL-6/CRP biomarkers	Not applicable	Median trial follow-up	Cardiovascular events
Ridker et al. (2017) [16]	Randomised controlled trial; atherosclerotic cardiovascular disease	Canakinumab/inflammation	Not applicable	Median 3.7 years	Recurrent cardiovascular events
Ohtsuka et al. (2001) [17]	Prospective clinical intervention study; dilated cardiomyopathy	Beta-blockade/cytokines	Not applicable	Not reported	No long-term clinical outcomes (short-term biomarker study)
Rezaie-Majd et al. (2002) [18]	Mechanistic clinical study; hypercholesterolaemia	Simvastatin/monocyte cytokines	Not applicable	Short treatment period	No long-term clinical outcomes (short-term biomarker study)
Khuri et al. (2005) [19]	Prospective multicentre cohort; cardiac surgery	Regional myocardial acidosis	Not reported	Long-term follow-up	Long-term survival
Deitch 2012 [20]	Mechanistic review; critical illness/sepsis	Gut-origin sepsis	Not applicable	Not applicable	No long-term clinical outcomes (mechanistic review)
Carré et al. (2010) [21]	Prospective translational cohort; critical illness	Mitochondrial biogenesis	Not applicable	ICU course	Survival
Singer (2014) [22]	Mechanistic review; sepsis/multi-organ failure	Mitochondrial dysfunction	Not applicable	Not applicable	No long-term clinical outcome (mechanistic review)
Hirakawa et al. (2005) [30]	Basic/translational animal study; tumour biology	VEGF-A/lymphangiogenesis	Not applicable	Experimental follow-up	Lymphatic metastasis
Abramovitch et al. (1999) [31]	Basic/translational animal study; tumour biology	Wound-derived growth factors	Not applicable	Experimental follow-up	Tumour growth
Alonso et al. (2015) [32]	Prospective matched cohort; colorectal cancer resection	Intra-abdominal infection	Clinical infection definitions	Long-term follow-up	Cancer recurrence
Yu et al. (2010) [33]	Prospective translational clinical study; gastric cancer surgery	Surgical inflammatory response	Not applicable	Early postoperative period	No long-term clinical outcomes (short-term translational biomarker study)
Hayashi et al. (2007) [48]	In vivo imaging translational study; tumour biology	Tumour cell lymphatic trafficking	Not applicable	Experimental follow-up	Cell shedding/dissemination
Echizen et al. (2016) [57]	Mechanistic review; gastric cancer	COX-2/PGE2 and TLR/MyD88	Not applicable	Not applicable	No long-term clinical outcome (mechanistic review)
Soumaoro et al. (2004) [59]	Retrospective/prognostic cohort; colorectal cancer	COX-2 expression	Immunohistochemical expression	Long-term follow-up	Prognosis/survival
Chen et al. (2011) [61]	In vitro translational study; cancer cell biology	Celecoxib/c-FLIP degradation	Not applicable	Experimental duration	Apoptosis signalling
Wei et al. (2004) [62]	In vitro/animal translational study; pancreatic cancer	Celecoxib/VEGF suppression	Not applicable	Experimental follow-up	Angiogenesis and metastasis
Menger and Vollmar (2004) [74]	Mechanistic review; surgical trauma	Hyperinflammation vs immunosuppression	Not applicable	Not applicable	No long-term clinical outcomes (mechanistic review)
Jawa et al. (2011) [75]	Narrative review; surgery, trauma, and critical care	IL-6	Not applicable	Not applicable	No long-term clinical outcomes (mechanistic review of IL-6)
López-Cuenca et al. (2013) [77]	Prospective observational cohort; non-ST-elevation acute coronary syndromes	IL-6 and hs-CRP	Not applicable	Long-term follow-up	Cardiovascular outcomes
Reniers et al. (2025) [78]	Prospective cohort; pancreatic surgery	Preoperative IL-6/chronic inflammation	Perioperative myocardial injury definition (hs-cTnT rise)	48-hour biomarker follow-up	No long-term clinical outcomes (perioperative biomarker and injury endpoints only)
Bohle et al. (2010) [79]	Basic/translational mouse study; colon cancer excision	Postoperative intra-abdominal infection	Not applicable	Experimental follow-up	Angiogenesis; tumour recurrence
Wang et al. (2015) [80]	Basic/translational mouse study; colorectal cancer	PGE2	Not applicable	Experimental follow-up	Cancer stem cell expansion; metastasis
Finetti et al. 2020 [81]	Mechanistic review; cancer biology	PGE2	Not applicable	Not applicable	No long-term clinical outcomes (mechanistic review)
Sheehan et al. (1999) [82]	Translational prognostic tissue study; colorectal cancer	COX-2 expression	Immunohistochemical expression	Not reported	Association with tumour biology/prognosis
Xiong et al. (2003) [83]	Translational tissue study; colorectal cancer	COX-2 expression and angiogenesis	Immunohistochemical expression	Not reported	Angiogenesis association
Desmedt et al. (2018) [84]	Retrospective cohort/secondary analysis; breast cancer surgery	Intraoperative ketorolac	Not applicable	Long-term follow-up	Breast cancer recurrence
Neuroendocrine stress signalling (n=17)					
Desborough (2000) [35]	Mechanistic review; trauma and surgery	Neuroendocrine stress response	Not applicable	Not applicable	No long-term clinical outcome (mechanistic review)
Bernabe et al. (2011) [37]	In vitro translational study; oral squamous cell carcinoma	Stress hormones	Not applicable	Experimental duration	Cell proliferation/IL-6 secretion
Sloan et al. (2010) [38]	Basic/translational animal study; breast cancer	Sympathetic activation	Not applicable	Experimental follow-up	Metastatic switch
Kim-Fuchs et al. (2014) [39]	Basic/translational animal study; pancreatic cancer	Chronic stress/beta-adrenergic signalling	Not applicable	Experimental follow-up	Tumour growth and invasion
Masur et al. (2001) [40]	In vitro translational study; colon carcinoma	Norepinephrine/migration	Not applicable	Experimental duration	Cell migration
Wolter et al. (2014) [41]	Preclinical translational study; neuroblastoma	Propranolol	Not applicable	Experimental follow-up	Tumour growth/antitumour activity
Thaker et al. (2006) [42]	Basic/translational animal study; ovarian carcinoma	Chronic stress	Not applicable	Experimental follow-up	Tumour growth and angiogenesis
Magnon et al. (2013) [43]	Basic/translational animal study; prostate cancer	Autonomic innervation	Not applicable	Experimental follow-up	Cancer progression
Yang et al. (2009) [44]	In vitro translational study; melanoma	Norepinephrine	Not applicable	Experimental duration	VEGF/IL-8/IL-6 expression
Yang et al. (2006) [45]	In vitro translational study; nasopharyngeal carcinoma	Norepinephrine	Not applicable	Experimental duration	VEGF/MMP expression
Creed et al. (2015) [46]	In vitro translational study; breast cancer	Beta2-adrenoceptor signalling	Not applicable	Experimental duration	Invasion/invadopodia
Pon et al. (2016) [47]	In vitro translational study; breast cancer	Beta2-adrenoceptor signalling	Not applicable	Experimental duration	Cell invasion
Hiller et al. (2016) [49]	First-in-human proof-of-concept study; perioperative physiology	Neuraxial anaesthesia/lymphatic flow	Not applicable	Perioperative	Lymphatic flow; no oncologic outcome
Le et al. (2016) [50]	Basic/translational animal study; tumour biology	Chronic stress/lymphatic remodelling	Not applicable	Experimental follow-up	Tumour dissemination
Campbell et al. (2012) [51]	Basic/translational animal study; breast cancer	Sympathetic nerves/bone marrow stroma	Not applicable	Experimental follow-up	Bone metastasis
Behrenbruch et al. (2018) [60]	Narrative/mechanistic review; colorectal cancer surgery	Surgical stress and metastasis	Not applicable	Not applicable	No long-term clinical outcomes (mechanistic review)
Elenkov and Chrousos (2002) [66]	Mechanistic review; stress/autoimmunity	Stress hormones and cytokines	Not applicable	Not applicable	No long-term clinical outcome (mechanistic review)
Perioperative immune dysregulation (n=17)					
Kloosterman et al. (1994) [9]	Prospective comparative clinical study; laparoscopic cholecystectomy	Laparoscopy/immune function	Not applicable	Short postoperative sampling	No long-term clinical outcomes (short-term immune/physiologic study)
Gaudillière et al. (2014) [14]	Prospective translational cohort; surgery	Single-cell immune signatures	Not applicable	Postoperative recovery period	No long-term clinical outcomes (short-term clinical recovery/immune signature study)
Netea et al. (2016) [23]	Mechanistic review; innate immunity	Trained immunity	Not applicable	Not applicable	No long-term clinical outcomes (mechanistic review)
Curtis et al. (2003) [24]	Mechanistic review; perioperative oncology	Antitumour immunity	Not applicable	Not applicable	No long-term clinical outcomes (mechanistic review)
Jackson and Rice (1990) [27]	Retrospective cohort; head and neck cancer surgery	Wound infection	Not reported	Long-term follow-up	Cancer recurrence
Tang et al. (2020) [29]	Narrative review; cancer surgery	Surgical trauma immunosuppression	Not applicable	Not applicable	No long-term clinical outcomes (mechanistic review)
Hiller et al. (2018) [36]	Narrative/mechanistic review; cancer surgery	Perioperative events and recurrence	Not applicable	Not applicable	No long-term clinical outcomes (mechanistic review of recurrence risk)
Benish et al. (2008) [52]	Preclinical perioperative intervention study; cancer surgery model	Beta-blocker+COX-2 inhibition	Not applicable	Experimental follow-up	Immune competence; metastasis
Goldfarb et al. (2011) [53]	Preclinical perioperative intervention study; cancer surgery model	Postoperative immune status	Not applicable	Experimental follow-up	Resistance to metastasis
Hüttner et al. (2025) [54]	Phase II randomised controlled trial; pancreatic resection	Propranolol+etodolac	Not reported	Protocol follow-up	Perioperative/oncologic feasibility outcomes
Dubowitz et al. (2021) [55]	Feasibility/pilot randomised trial; cancer surgery	Volatile anaesthesia	Not reported	Pilot follow-up	Feasibility; perioperative cancer-related outcomes
Ricon et al. (2019) [56]	Narrative review; perioperative oncology	Beta-adrenergic+COX-2 inhibition	Not applicable	Not applicable	No long-term clinical outcomes (mechanistic review of recurrence prevention)
Heusinkveld et al. (2011) [58]	Translational immunology study; cervical carcinoma	PGE2/IL-6-driven macrophage polarisation	Not applicable	Experimental duration	No long-term clinical outcomes (experimental mechanistic study)
Forget et al. (2010) [63]	Retrospective cohort; mastectomy for breast cancer	Intraoperative analgesics	Not reported	Long-term follow-up	Breast cancer recurrence
Hogan et al. (2011) [64]	Narrative review; surgery	Surgery-induced immunosuppression	Not applicable	Not applicable	No long-term clinical outcomes (mechanistic review)
Sietses et al. (1999) [65]	Narrative/mechanistic review; laparoscopic surgery	Immunological consequences of laparoscopy	Not applicable	Not applicable	No long-term clinical outcomes (mechanistic review)
Bohne et al. (2024) [76]	Systematic review and meta-analysis; colorectal cancer surgery	Laparoscopic vs open surgery/humoral immunity	Not applicable	Variable	Humoral immune outcomes; no clear long-term survival endpoint
Disruption to adjuvant treatment pathways (n=11)					
Mirnezami et al. (2011) [25]	Systematic review and meta-analysis; colorectal cancer resection	Anastomotic leak	Study-specific	Variable	Local recurrence; overall survival
Andalib et al. (2013) [26]	Population-based retrospective cohort; lung cancer surgery	Postoperative infectious complications	Administrative/clinical definitions	Long-term follow-up	Overall survival
Hayashi et al. (2015) [28]	Retrospective cohort; gastric cancer surgery	Infectious complications	Not reported	Long-term follow-up	Cancer recurrence
Indelicato et al. (2007) [34]	Retrospective cohort; breast-conserving therapy	Acute infection/closure type	Clinical wound infection	Long-term surveillance	Local recurrence
Notarfrancesco et al. (2023) [67]	Retrospective chart review; post-chemotherapy retroperitoneal lymph node dissection	Perioperative complications	Not reported	Oncological follow-up	Oncological outcomes/recurrence
Yamashita et al. (2017) [68]	Retrospective prognostic cohort; resection of colorectal liver metastases	Postoperative complication burden	CCI	Long-term follow-up	Cancer-specific survival
Mavros et al. (2013) [69]	Retrospective cohort; colorectal liver metastasis resection	Postoperative complications	Not reported	Long-term follow-up	Overall survival
Murthy et al. (2007) [70]	Population-based cohort; breast cancer surgery	Postoperative wound complications	Clinical wound complication definitions	Long-term follow-up	Systemic recurrence
Broecker et al. (2017) [71]	Retrospective cohort; truncal/extremity soft-tissue sarcoma resection	Postoperative complications	Not reported	Long-term follow-up	Oncologic outcomes
Boukovalas et al. (2020) [72]	Retrospective cohort; total laryngectomy	Postoperative complications	Not reported	Up to 10 years	Oncologic outcomes
Krarup et al. (2014) [73]	Nationwide retrospective cohort; curative colonic cancer resection	Anastomotic leak	Clinical/anastomotic leak definitions	Long-term follow-up	Distant recurrence; long-term mortality

Overview of Mechanistic Domains

Across these studies, four recurrent mechanistic domains emerged. First, postoperative complications appeared to amplify and prolong systemic inflammation, with potential downstream effects on endothelial function, cardiovascular risk, organ dysfunction, and tissue repair [8-23]. Second, in oncological surgery, postoperative complications were associated with biological conditions that may favour tumour cell survival, angiogenesis, dissemination, and metastatic niche formation [24-63]. Third, perioperative immune dysregulation, particularly the suppression of natural killer-cell and cytotoxic T-cell activity, may impair control of infection and minimal residual disease [64-67]. Fourth, in selected cancer populations, major postoperative complications may delay, interrupt, or preclude the timely delivery of adjuvant therapy [68-73]. Taken together, these domains suggest that postoperative complications may function not only as markers of severe illness but also as biologically consequential events with the potential to influence long-term prognosis, although the extent of any causal contribution remains uncertain.

Persistent Systemic Inflammation

Surgical trauma triggers a systemic inflammatory response characterised by the release of proinflammatory cytokines, including tumour necrosis factor α and interleukin (IL)-1β, followed by the downstream induction of IL-6 and acute-phase proteins such as C-reactive protein (CRP) [10,74]. The magnitude of this response appears to correlate with the extent of surgical injury, with lower IL-6 concentrations observed after laparoscopic than open procedures, suggesting that the nature of the operative insult partly determines the postoperative inflammatory trajectory [75,76]. In uncomplicated recovery, this response is usually self-limited. By contrast, postoperative complications, particularly sepsis, pneumonia, and wound infection, appear to intensify and prolong inflammation beyond the expected recovery period [10-12].

Several studies support the clinical relevance of this amplified inflammatory state. Higher postoperative inflammatory burden, as reflected by early postoperative CRP and IL-6 concentrations, has been associated with major complications, delayed recovery, persistent disability, and death after major surgery [10-12,77,78]. Translational studies further suggest that early dysregulation of innate immune pathways may identify patients most vulnerable to this trajectory [13,14]. Upregulation of Toll-like receptor and nuclear factor kappa-light-chain-enhancer of activated B cell (NF-κB) signalling in circulating monocytes has been associated with the subsequent development of systemic inflammatory response syndrome after major abdominal surgery, while single-cell immune profiling has linked the activation of pathways such as signal transducer and activator of transcription 3 (STAT3), cAMP response element-binding protein (CREB), and NF-κB with delayed functional recovery after orthopaedic surgery [13,14]. These observations support the concept that postoperative complications do not merely coexist with systemic inflammation, but may amplify a maladaptive host response.

Persistent systemic inflammation provides one plausible mechanism by which postoperative complications could influence long-term cardiovascular and organ-specific outcomes [15,16,77,78]. Sustained elevation of IL-6 and CRP has been associated with increased risks of myocardial infarction and all-cause mortality in broader clinical populations, supporting the biological plausibility that prolonged inflammatory activation after surgery may contribute to endothelial dysfunction, subclinical myocardial injury, and accelerated atherosclerosis [15,16]. This concept is reinforced by the Canakinumab Anti-inflammatory Thrombosis Outcomes Study (CANTOS) trial, in which IL-1β inhibition reduced recurrent cardiovascular events independently of lipid lowering, indicating that inflammatory signalling can directly influence long-term vascular risk [16]. Additional evidence suggests that therapies with anti-inflammatory effects, including β-blockers and statins, may modulate cytokine production and endothelial activation, offering a potential mechanistic bridge between perioperative inflammation and later cardiovascular outcomes [17,18].

Experimental and translational data also suggest that severe or prolonged inflammatory states may contribute to broader physiological deterioration. In cardiac surgery, intraoperative myocardial acidosis has been associated with reduced long-term survival, raising the possibility that inflammation-related tissue stress, apoptosis, and impaired cellular recovery may contribute to later adverse outcomes [19]. Sustained systemic inflammation may also perpetuate downstream injury through gastrointestinal barrier dysfunction, bacterial translocation, mitochondrial impairment, and epigenetic reprogramming of innate immunity [20-23]. Although some of these pathways remain incompletely validated in perioperative populations, together they support a model in which postoperative complications act as amplifiers of systemic inflammation, thereby creating a biological milieu less compatible with long-term recovery and survival [19-23].

Postoperative Complications and Tumour Progression

In oncological surgery, postoperative complications may influence long-term outcome not only through general physiological deterioration but also through direct effects on tumour biology [24-29]. Following the resection of a primary tumour, control of minimal residual disease is critical to preventing recurrence and metastatic spread. Across several malignancies, including colorectal, gastro-oesophageal, lung, and head and neck cancers, infectious postoperative complications have been associated with increased recurrence and poorer survival [25-28]. The mechanistic basis of this association is likely multifactorial, involving proinflammatory signalling, sympathetic activation, and prostaglandin-mediated changes in the tumour microenvironment [29-63].

Proinflammatory Signalling and Angiogenesis

Postoperative infection can intensify the inflammatory response and increase the release of soluble growth factors such as vascular endothelial growth factor and matrix metalloproteinases, both of which promote angiogenesis, cellular proliferation, and tumour propagation [30-32,79]. In colorectal cancer, anastomotic leak and postoperative peritoneal infection have been associated with higher circulating IL-6 and vascular endothelial growth factor concentrations, together with increased local recurrence and poorer survival [25,32]. Prospective data also suggest that postoperative intra-abdominal infection may generate inflammatory and angiogenic conditions that favour survival of residual cancer cells within the operative field, venous circulation, or occult micro-metastatic deposits [31,32].

Proinflammatory cytokines may also enhance adhesion and colonisation of circulating tumour cells; for example, IL-1 and tumour necrosis factor-alpha (TNF-α) have been implicated in facilitating the adhesion of gastric cancer cells to mesothelial surfaces, while acute breast infection after breast-conserving surgery has been associated with increased local recurrence [33,34]. These findings support the view that infectious complications may do more than indicate a difficult postoperative course; they may contribute to a biological environment permissive to tumour persistence and recurrence [25,31-34].

Sympathetic Activation and Metastatic Dissemination

The perioperative stress response represents a second mechanism by which postoperative complications may promote tumour progression [35-37]. Surgical trauma, patient anxiety, fasting, hypothermia, and metabolic disturbance activate the sympathetic nervous system, and more extensive operations or complicated postoperative courses are associated with higher circulating catecholamine concentrations [35-37]. Catecholamines have been shown to stimulate tumour cell proliferation and to promote a microenvironment conducive to metastatic deposition [37-45]. Across several preclinical cancer models, β-adrenergic signalling enhances angiogenesis, migration, invasion, and metastatic behaviour in breast, pancreatic, colon, ovarian, and prostate cancer and neuroblastoma [38-43].

Mechanistically, β-adrenergic activation increases the tumour expression of vascular endothelial growth factor, matrix metalloproteinases, IL-6, and IL-8 and induces structural changes that facilitate cell invasion and metastatic competence [44-47]. Catecholamine signalling may also remodel lymphatic and blood vasculature, accelerate lymphatic flow, and condition distant tissues to become receptive metastatic niches [48-51]. These pathways are particularly relevant to postoperative complications because inflammatory and physiological stress responses often coexist and may potentiate one another. Although much of this evidence is preclinical, inhibition of sympathetic signalling with β antagonists such as propranolol has reduced tumour invasion, angiogenesis, lymphangiogenesis, epithelial-to-mesenchymal transition, and postoperative metastatic susceptibility in experimental studies [40,41,52,53]. Early translational trials, including perioperative β-blockade strategies and feasibility studies of anaesthetic modulation, have therefore begun to test whether this biology can be therapeutically targeted in the perioperative period [54-56].

Prostaglandin Pathways

Prostaglandin signalling provides a further mechanistic link between postoperative complications and tumour progression. Postoperative complications may increase prostaglandin E2 concentrations both locally and systemically, thereby sustaining inflammation and promoting tumour cell stemness, invasiveness, and angiogenesis [80,81]. Prostaglandin E2 has also been shown to skew macrophage differentiation towards tumour-promoting phenotypes and to support tumour angiogenesis. In colorectal cancer, cyclooxygenase-2 expression correlates with tumour size, stage, vascularisation, nodal involvement, recurrence, and overall survival, suggesting that prostaglandin-mediated pathways may be clinically relevant determinants of tumour behaviour [81-83]. Experimental inhibition of cyclooxygenase-2 reduces proangiogenic signalling and tumour microvascular density and promotes tumour cell apoptosis. In addition, intraoperative ketorolac use during breast cancer surgery has been associated with reduced recurrence [63,84], providing clinical support for the hypothesis that perioperative prostaglandin suppression may influence longer-term oncological outcomes.

Taken together, these findings suggest that postoperative complications may help generate a transient but biologically important perioperative window in which inflammatory and neuroendocrine perturbations facilitate tumour survival and dissemination [24-63]. Although the relative contribution of each pathway is uncertain and much of this evidence is preclinical, the convergence of clinical, translational, and experimental findings supports the biological plausibility that complicated postoperative recovery may contribute to later cancer recurrence in selected settings [25-63].

Suppression of Antitumour Immunity

Perioperative immunosuppression is a well-described consequence of surgical stress and provides another plausible pathway linking postoperative complications with adverse long-term oncological outcomes [64-67]. Activation of the hypothalamic-pituitary-adrenal axis and sympathetic nervous system after surgery leads to the release of glucocorticoids, catecholamines, and cytokines that suppress cell-mediated immunity [35,36,64-66]. This response is associated with reduced number and activity of natural killer cells and activated CD8-positive T cells, expansion of regulatory T-cell populations, and a shift from antitumour T-helper-1 responses towards more tumour-promoting T-helper-2 profiles [64-66]. The extent of these changes appears to correlate with the magnitude of the surgical insult [8,9,64,65].

In the setting of postoperative complications, this window of immune vulnerability may be prolonged or intensified [64-67]. Experimental studies have shown that perioperative immune suppression increases postoperative metastatic disease, while clinical and translational studies link similar immune alterations with recurrence and mortality across multiple tumour types [24,29,64-67]. These findings suggest that postoperative complications may impair host control of minimal residual disease not only through inflammation and stress signalling but also through diminished immune surveillance [65,67]. Although the relative contribution of immune dysregulation compared with other pathways remains difficult to quantify, the consistency of this biological theme across the literature supports its inclusion as a major mechanistic domain [66,67].

Disruption to Adjuvant Treatment Pathways

A further explanation for poorer long-term outcomes after postoperative complications, particularly in cancer surgery, is disruption to the timely delivery of adjuvant treatment [68-73]. Major complications are associated with prolonged intensive care and hospital stay, increased reoperation, and higher readmission rates, all of which may delay functional recovery and reduce fitness for chemotherapy or radiotherapy [4,68-73]. Because timely adjuvant treatment may suppress residual disease and mitigate postoperative biological vulnerability, delays or omission of therapy represents a clinically plausible pathway through which complications could worsen long-term oncological outcomes [68-73].

However, the evidence in this area is mixed. Some studies in patients with colorectal liver metastases, breast cancer, and soft-tissue sarcoma have found no clear difference in the initiation of adjuvant therapy between patients with and without postoperative complications or between those with minor and major complications [69-71]. By contrast, in other tumour types, postoperative complications and delays to adjuvant therapy have each been associated with inferior disease-free and overall survival [70-73]. In total laryngectomy cohorts, both factors independently predicted worse outcomes [72]. Similarly, in stage III colon cancer, anastomotic leak was associated with reduced likelihood of receiving adjuvant chemotherapy and with longer delay to treatment initiation among those who did receive therapy [73]; this was accompanied by reduced overall survival, although distant recurrence was not significantly different.

**Figure 2:**
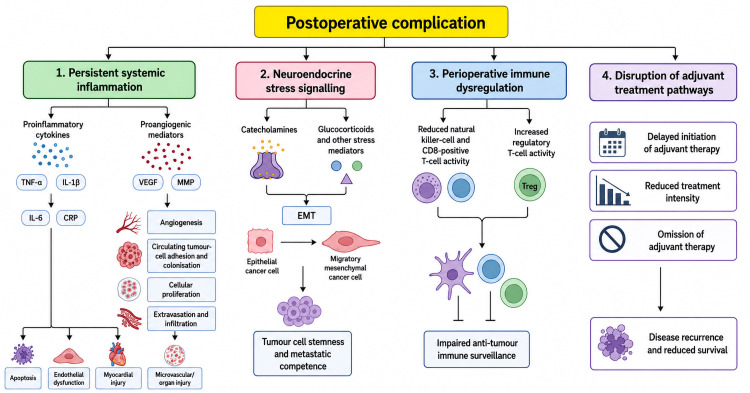
Proposed pathways by which postoperative complications may influence long-term outcomes. Graphical images were created using Microsoft PowerPoint (Microsoft 365; Microsoft Corporation, Redmond, WA, USA) and edited in Adobe Photoshop 2026 (version 27.7; Adobe Inc., San Jose, CA, USA) TNF‑α: tumour necrosis factor alpha; IL-1β: interleukin-1 beta; IL‑6: interleukin‑6; VEGF: vascular endothelial growth factor; MMP: matrix metalloproteinase; CRP: C‑reactive protein; EMT: epithelial-mesenchymal transition; Treg: regulatory T cell

These observations suggest that disruption to adjuvant therapy is not a universal mechanism, but may be particularly relevant in selected tumour types and stages [68-73]. In colorectal and stage III colon cancer, anastomotic leak and major complications have been associated with delayed or reduced receipt of adjuvant chemotherapy and with poorer survival in some cohorts. In breast cancer and soft-tissue sarcoma, the relationship appears less consistent. In head and neck cancer, including total laryngectomy cohorts, postoperative complications and delayed adjuvant therapy have both been associated with inferior oncological outcomes. Accordingly, treatment disruption should be interpreted as a context-dependent pathway rather than a uniform explanation for the long-term impact of postoperative complications across all cancers [68-73]. A graphical overview of the proposed pathways is presented in Figure 2.

Discussion

Principal Findings

This narrative review confirms a consistent association between postoperative complications and impaired long-term survival across both oncological and non-oncological surgery, an effect that is unlikely to be explained solely by operative severity or baseline frailty [5-7]. Across diverse study designs, four recurrent and biologically plausible mechanistic domains emerge: persistent systemic inflammation, exaggerated neuroendocrine stress signalling, perioperative immune dysregulation, and, in selected cancer populations, disruption to the delivery of adjuvant therapy [8-29,35-73]. Taken together, these domains support the interpretation that postoperative complications may act not only as markers of perioperative adversity but also as biological amplifiers with the potential to influence long-term prognosis; however, the current evidence base is heterogeneous and does not establish definitive causality [8-29,35-73].

Biological and Clinical Implications

Among these domains, persistent systemic inflammation appears central to linking postoperative morbidity with later cardiovascular and organ-specific outcomes [8-23]. Surgical trauma initiates a coordinated inflammatory response that is normally self-limited, but postoperative complications, particularly infection, sepsis, and extensive tissue injury, can intensify and prolong this response [8-14]. Sustained elevations of mediators such as IL-6 and CRP provide a plausible pathway through which complications extend their effects beyond the index admission, promoting endothelial dysfunction, atherothrombotic risk, maladaptive tissue repair, and progressive organ injury [10-19]. Experimental and translational data further implicate inflammatory-driven mitochondrial dysfunction, trained innate immunity, and cellular senescence in creating a persistent biological milieu less compatible with physiological resilience and long-term survival [19-23].

The interaction between postoperative complications and tumour biology represents a second major theme [24-63]. In several malignancies, infectious and inflammatory complications are associated with higher recurrence rates and poorer survival, consistent with a model in which postoperative events may amplify processes that favour residual tumour survival, angiogenesis, invasion, lymphatic dissemination, and metastatic niche formation [25-34]. Proinflammatory cytokines, growth factors, β-adrenergic signalling, and prostaglandin pathways appear to converge on tumour cells and the host microenvironment in ways that are biologically plausible and mechanistically coherent [30-32,35-63]. However, much of this evidence remains translational or preclinical, and the magnitude and consistency of any causal effect in specific tumour types remain uncertain [24-63]. Perioperative immune dysregulation provides a further mechanistic bridge between postoperative complications and poor long-term outcomes [64-67]. Surgical stress suppresses cell-mediated immunity, reduces natural killer-cell and cytotoxic T-cell activity, expands regulatory T-cell populations, and shifts cytokine profiles towards more tumour-permissive phenotypes [64-66]. In the setting of postoperative complications, this period of immune vulnerability may be prolonged or intensified, potentially weakening host control of infection, impairing tissue recovery, and reducing surveillance of minimal residual malignant disease [64-67]. Although the independent contribution of these immunological changes to long-term mortality is difficult to quantify, their consistency across experimental and translational studies suggests that immune perturbation is an important component of the broader biological response to complicated recovery [64-67].

The effect of postoperative complications on adjuvant treatment delivery appears more context-dependent but remains clinically relevant [68-73]. Major complications can prolong hospitalisation, increase readmission and reoperation, and delay functional recovery, thereby compromising the initiation, intensity, or completion of adjuvant therapy. The downstream impact of this disruption varies by tumour type, disease stage, and complication severity, with some studies showing minimal effect and others demonstrating clear associations with worse disease-free or overall survival [69-73]. This heterogeneity implies that treatment disruption is unlikely to be a universal explanation for the long-term impact of postoperative complications, but in selected cancer populations, it may represent an additional and potentially modifiable pathway through which perioperative morbidity influences prognosis [69-73].

Strengths and Limitations

The strength of this review lies in its integrative mechanistic framework, which draws together epidemiological, translational, and experimental evidence to provide a more coherent account of how postoperative complications might influence long-term cardiovascular, oncological, and organ-specific outcomes [5-73]. By synthesising across domains that are usually considered separately, including systemic inflammation, perioperative stress biology, immune dysfunction, and cancer recurrence, the review highlights shared pathways that may be amenable to targeted perioperative interventions [8-29,35-73].

Several limitations should be recognised. First, this is a narrative review informed by structured searches rather than a formal systematic review; although eligibility criteria, database searches, and a Preferred Reporting Items for Systematic Reviews and Meta-Analyses (PRISMA)-style flow diagram were used, protocol registration, formal risk-of-bias assessment, formal evidence grading, and a full systematic review workflow were not undertaken. Second, the synthesis deliberately combines observational clinical studies with translational and preclinical evidence, which broadens mechanistic insight but limits direct inference regarding causality, effect size, and generalisability to specific patient populations. Third, important heterogeneity exists across surgical populations, complication definitions, severity classifications, follow-up duration, and long-term outcome measures, which precluded meta-analysis and limits direct comparison between studies [3,5-7,68-73]. Fourth, although the review focuses on four recurrent biological domains, other emerging mechanisms may also be relevant and were not comprehensively captured [8-73]. These limitations should be considered when interpreting the review as an integrative mechanistic framework rather than as a definitive quantitative estimate of the long-term effects of postoperative complications.

Future Directions

Current evidence supports a biologically plausible framework for the adverse long-term associations observed after postoperative complications, but important uncertainties remain regarding the relative contribution and interaction of inflammatory, neuroendocrine, immune, and treatment-disruption pathways across different surgical populations and tumour types [8-73]. Future research should prioritise biomarker-rich prospective perioperative cohorts, tumour-specific and complication-specific analyses, and interventional trials designed to test whether modulation of these pathways can alter long-term outcomes [11-18,52-56,61-63,68-73].

Large, biomarker-rich perioperative cohorts incorporating inflammatory markers, immune phenotyping, and neuroendocrine profiling would help clarify how specific biological responses track with complication severity, cardiovascular events, cancer recurrence, and overall mortality [11-23,64-67]. Randomised trials are needed to determine whether perioperative modulation of these pathways can alter long-term outcomes; promising strategies include β-adrenergic blockade, cyclooxygenase-2 inhibition, and other anti-inflammatory or immunomodulatory interventions explicitly designed around mechanistic as well as clinical endpoints [16-18,52-56,61-63]. In oncological surgery, tumour- and stage-specific studies should elucidate how postoperative complications interact with minimal residual disease, immune escape, and the timing and effectiveness of adjuvant therapy, potentially incorporating minimal residual disease assays and longitudinal immune monitoring [24-29,68-73]. Beyond intervention studies, implementation and health service research will be required to establish whether perioperative care models that reduce biological stress and complication burden can be scaled to deliver equitable and cost‑effective improvements in long‑term survival and patient‑centred outcomes.

## Conclusions

Postoperative complications are consistently associated with impaired long‑term survival and major adverse outcomes after both oncological and non-oncological surgery. The evidence synthesised in this review suggests that this association is biologically plausible and likely multifactorial, involving interacting pathways of persistent systemic inflammation, neuroendocrine stress signalling, and perioperative immune dysregulation, with additional contributions from disruption to adjuvant treatment in selected cancer populations. These mechanisms provide a coherent framework through which postoperative complications may influence cardiovascular risk, organ dysfunction, tumour progression, and ultimately long-term survival.

This perspective reframes postoperative complications as more than isolated perioperative events or markers of patient frailty; rather, they represent biologically consequential episodes that amplify downstream pathological processes during a vulnerable perioperative window. Recognising this broader significance has important implications for perioperative medicine, because strategies that prevent complications or attenuate their inflammatory, neuroendocrine, and immune consequences might improve not only short-term recovery but also longer-term outcomes. Although the current evidence base remains incomplete, particularly with respect to causality, the relative contribution of different pathways in specific populations, and the extent to which biological modulation can alter prognosis, the mechanistic signals are sufficiently compelling to justify a more integrated research agenda spanning biomarker-rich cohort studies, translational investigations, and randomised trials designed around both mechanistic and long-term clinical endpoints. Viewed in this way, the perioperative period is not simply a short interval of technical risk but a potential therapeutic window in which biologically informed interventions could meaningfully shape survival after surgery.

## Appendices

Database search strategies

The original structured database searches were conducted in Ovid MEDLINE and Embase (Excerpta Medica dataBASE) (via Ovid) from database inception to November 30, 2025. A subsequent focused Ovid MEDLINE update search was undertaken from December 1, 2025, to March 31, 2026, to identify newly published mechanistic and perioperative studies relevant to the review question. The original database search records contributed to the primary Preferred Reporting Items for Systematic Reviews and Meta-Analyses (PRISMA) screening set, and the focused MEDLINE update did not identify any additional eligible studies beyond those already captured in the original search.

**Table 3:** Ovid MEDLINE database search strategies Searches were performed in Ovid MEDLINE and Embase via Ovid using a combination of controlled vocabulary terms and free-text keywords to maximise sensitivity. The original structured database searches were conducted from database inception to November 30, 2025, in both databases, and a subsequent focused Ovid MEDLINE update was performed from December 1, 2025, to March 31, 2026. In Ovid syntax, a trailing slash "/" denotes a controlled vocabulary subject heading (MeSH in MEDLINE, Emtree in Embase). Field tags specify where terms were searched within each record: ".ti" indicates the title field, ".ab" the abstract field, and ".kf" the author keyword field; ".ti,ab,kf." denotes a combined search across all three fields. Free-text terms were searched in these fields to capture records that might not yet have been indexed under the relevant subject headings. The asterisk "*" was used as a truncation symbol to retrieve variant word endings, including singular/plural forms and related derivations (e.g., "complication", "outcome*", "fatalit*", "cytokine*", "immunosuppress*", and "pathophysiolog*"). Boolean operators structured the search: "OR" combined synonyms, spelling variants, and closely related terms within each concept block, whereas "AND" combined separate concept blocks so that retrieved records addressed all required concepts. Hyphenated and non-hyphenated variants (e.g., "post-operative" and "postoperative" and "long-term" and "longterm") were searched separately to ensure comprehensive retrieval.

Ovid MEDLINE(R) ALL, 1946 to November 30, 2025 (original structured database search)	Number of papers
Postoperative Complications/	438,596
postoperative complication*.ti,ab,kf.	115,304
post-operative complication*.ti,ab,kf.	11,993
postsurgical complication*.ti,ab,kf.	1,367
post-surgical complication*.ti,ab,kf.	1,145
1 or 2 or 3 or 4 or 5	511,482
mortality/ or fatal outcome/ or survival rate/	319,334
Survival/	4,955
long-term mortality.ti,ab,kf.	12,330
longterm mortality.ti,ab,kf.	47
fatal outcome*.ti,ab,kf.	12,910
survival rate.ti,ab,kf.	131,505
long-term survival.ti,ab,kf.	53,208
longterm survival.ti,ab,kf.	326
long-term outcome*.ti,ab,kf.	93,592
longterm outcome*.ti,ab,kf.	457
long-term fatalit*.ti,ab,kf.	8
longterm fatalit*.ti,ab,kf.	0
7 or 8 or 9 or 10 or 11 or 12 or 13 or 14 or 15 or 16 or 17 or 18	571,099
(major surgery or cancer surgery).ti,ab,kf.	29,128
6 and 19 and 20	785
limit 21 to (english language and "humans only (removes records about animals)")	725
Total records	725
